# Sepsis, Endocarditis, and Purulent Arthritis due to a Rare Zoonotic Infection with *Streptococcus equi* Subspecies *zooepidemicus*

**DOI:** 10.1155/2018/3265701

**Published:** 2018-06-14

**Authors:** Anne Kirstine Høyer-Nielsen, Shahin Gaini, Anne Kjerulf, Rudi Kollslíð, Torkil Á Steig, Marc Stegger, Jan Jóanesarson

**Affiliations:** ^1^Division of Cardiology, Medical Department, National Hospital Faroe Islands, Tórshavn, Faroe Islands; ^2^Infectious Diseases Division, Medical Department, National Hospital Faroe Islands, Tórshavn, Faroe Islands; ^3^Infectious Diseases Research Unit, Odense University Hospital and University of Southern Denmark, Odense, Denmark; ^4^Centre of Health Research and Department of Science and Technology, University of the Faroe Islands, Tórshavn, Faroe Islands; ^5^Infectious Disease Epidemiology & Prevention, Infectious Disease Preparedness, Statens Serum Institut, Copenhagen, Denmark; ^6^Division of Nephrology, Medical Department, National Hospital Faroe Islands, Tórshavn, Faroe Islands; ^7^Bacteria, Parasites and Fungi, Infectious Disease Preparedness, Statens Serum Institut, Copenhagen, Denmark

## Abstract

*Streptococcus equi* subspecies *zooepidemicus* (*S. zooepidemicus*) is mostly known as an opportunistic pathogen found in horses and as a rare human zoonosis. An 82-year-old male, who had daily contact with horses, was admitted in a septic condition. The patient presented with dyspnea, hemoptysis, impaired general condition, and severe pain in a swollen left shoulder. Synovial fluid from the affected joint and blood cultures showed growth of *S. equi* subsp. *zooepidemicus*. Transesophageal echocardiography showed a vegetation on the aortic valve consistent with endocarditis. Arthroscopic revision revealed synovitis and erosion of the rotator cuff. Technetium-99m scintigraphy showed intense increased activity in the left shoulder, suspicious of osteitis. The infection was treated with intravenous antibiotics over a period of five weeks, followed by oral antibiotics for another two months. The patient recovered without permanent sequelae.

## 1. Introduction

*Streptococcus equi* subspecies *zooepidemicus* is classified as a beta-haemolytic, Lancefield group C streptococcal bacteria [1]. The bacterium is associated with opportunistic infections in horses, especially in the upper respiratory tract in horses, but can also cause infections in several other animals [1–3]. Transmission to humans is rare and is related to consumption of unpasteurized dairy products or contact with domestic animals, especially horses [1, 3]. Manifestation of the bacteria in humans is often associated with serious disseminated infections such as meningitis, glomerulonephritis, or sepsis [4], which also is the case in the present report. However, it can also be localised to one organ as pneumonia or purulent arthritis [5]. Previous reports of endocarditis and purulent arthritis due to *S. zooepidemicus* have only been documented in, respectively, 17 and 16 case reports (Tables 1 and 2). This is to our knowledge the first reported case of infective endocarditis in Scandinavia caused by *S. equi* subsp. *zooepidemicus*. We hereby seek to bring attention to *S. equi* subsp. *zooepidemicus* as a zoonotic pathogen, and the importance of clinical awareness on characteristic anamnestic information, hence animal contact when treating patients with sepsis or bacteraemia.

## 2. Case Presentation

An 82-year-old Caucasian male was admitted to our hospital in December 2016 with dyspnea, hemoptysis, and impaired general condition. He also presented with pseudoparalysis of his left shoulder due to severe pain. The medical record included ischemic heart disease (coronary artery bypass grafting in 1994), atrial fibrillation, low malignant prostate cancer, gout, and diabetes mellitus type II. Six months prior to admission, the patient had all teeth in his upper mouth removed prior to being fitted with dentures. This dental procedure was complicated with severe inflammation, and the patient was treated several times with oral antibiotics.

On admission, the patient was septic with fever and in a condition with pulmonary congestion and bilateral oedema in his lower limbs. Vital parameters included a blood pressure of 148/62 mmHg, a heart rate of 84 beats/min, oxygen saturation of 81% without oxygen supplementation, respiratory frequency at 26 per minute, and a rectal temperature of 38.8°C. Arterial blood gasses showed a normal pH (7.44), low partial pressure of carbon dioxide (3.5 kPa) and oxygen (7.2 kPa) in arterial blood, and low oxygen saturation (89%). On physical examination, the patient's left shoulder was tender and warm and had an anterior nonerythematous swelling. Cardiac auscultation did not reveal any murmur, and the neurologic examination was normal. The electrocardiogram revealed normofrequent atrial fibrillation and right bundle branch block.

The initial blood samples showed leucocytosis (14.7 × 10^9^/L) with dominance of neutrophilic granulocytes, haemoglobin level of 7.1 mmol/L, and C-reactive protein (CRP) of 216 mg/L.

Chest X-ray showed no infiltrates but was consistent with pulmonary stasis. An X-ray of the left shoulder showed no signs of inflammation.

**Figure 1:**
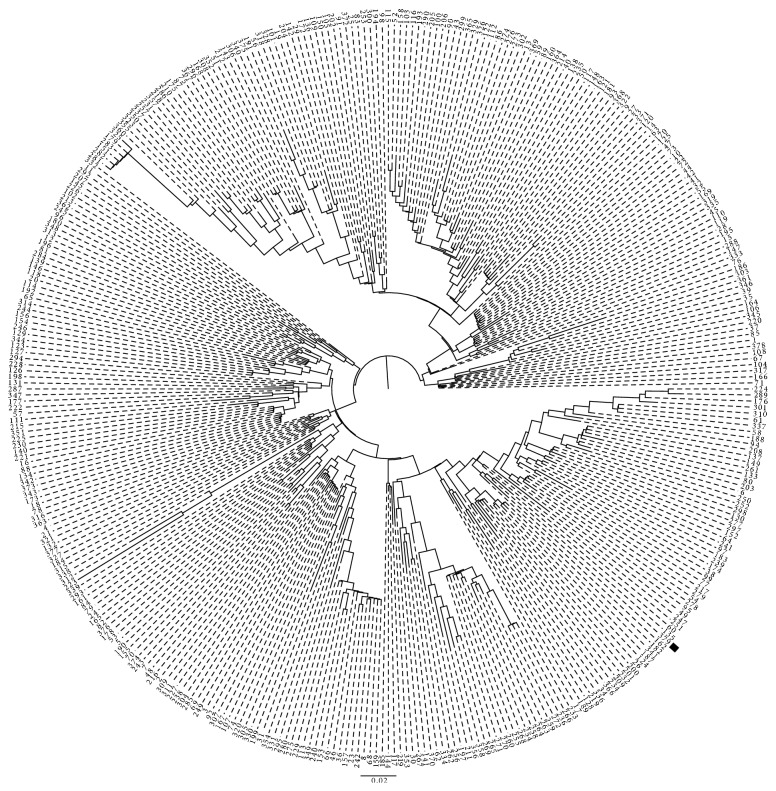
Relatedness of *Streptococcus equi* subsp. *zooepidemicus*. A midpoint-rooted phylogenetic analysis using a maximum likelihood-approximation of concatenated MLST allele for all publically available ST types, except ST's 248, 324 and 359 from which *yqiL* is partially lost. The location of ST379 is highlighted. The scale bar reflects genetic distance in expected number of nucleotide changes per site.

Blood cultures (three bottles with 10 ml each) and two samples of synovial fluids from the left shoulder were sent to the laboratory. Next day, growth of Gram-positive cocci was seen in one of the three blood culture bottles and in both synovial fluids. Matrix-assisted laser desorption ionization-time of flight mass spectrometry (MALDI-TOF MS) was performed on all three isolates using Microflex™ LT MALDI-TOF-System (Bruker, Karlsruhe, Germany; two databases: 1829023 Maldi Biotyper Compass Library and 8254705 Security ref. Library 1.0 for MALDI Biotyper 2.0), and they were diagnosed as *S. equi* subspecies *zooepidemicus* with a score between 2.37 and 2.63. Whole-genome sequencing of the blood culture isolate was performed by 251-bp paired-end sequencing (MiSeq; Illumina) and confirmed the species and subspecies type diagnosed by MALDI-TOF MS. The isolate carried a novel sequence type (ST379), and a phylogenetic analysis using FastTree v2.1.5 of the concatenated MLST alleles (Figure 1) found it to cluster in a branch containing ST60, ST135 and ST162. An analysis using the iTOL implementation at https://pubmlst.org/szooepidemicus/ revealed that seven related isolates with those ST types were reported in the database from various hosts (cow (*n*=1), dog (*n*=1), and horse (*n*=5)). The sequence data has been uploaded to ENA with the following Study and Read accession IDs: PRJEB24181 and ERR2233447, respectively. Antibiotic susceptibility testing using the Epsilometer test (E-test) (bioMérieux, Marcy-l'Etoile, France) showed that the isolate was susceptible to penicillin (0.016 microgram/ml), ampicillin (0.032 microgram/ml), amoxicillin (0.047 microgram/ml), cefuroxime (0.016 microgram/ml), erythromycin (0.094 microgram/ml), clindamycin (0.38 microgram/ml), vancomycin (0.5 microgram/ml), imipenem (0.012 microgram/ml), and rifampicin (0.012 microgram/ml). The isolate was intermediate susceptible to gentamicin (12 microgram/ml).

**Figure 2:**
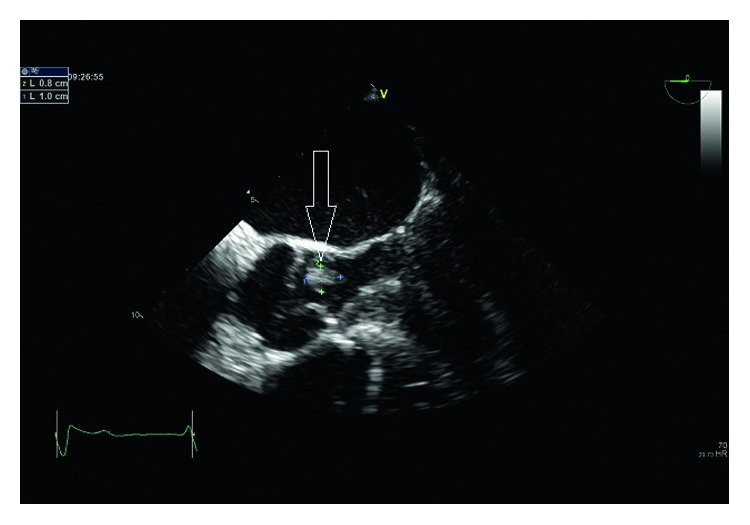
Illustration of the patient's transesophageal echocardiography demonstrating the vegetation on the aortic valve by the white arrow.

Antibiotic treatment the first two days was intravenous cefuroxime 1.5 gram administered three times daily, which was changed to intravenous high-dose benzylpenicillin, initially a dose of 2 million international units (IU) four times a day. The monotherapy with benzylpenicillin was supplemented with intravenous gentamicin in intervals (five days in total). On day three, transthoracic and transesophageal echocardiography (TTE and TEE, resp.) demonstrated a vegetation on the aortic valve (Figure 2) with a minimal aortic insufficiency. There were no signs of paravalvular abscesses and no stenosis of the affected valve. Ejection fraction was normal. The patient was diagnosed with aortic valve endocarditis without indication for acute operation. According to the national Danish guidelines for infective endocarditis, the benzylpenicillin dosage was increased to 5 million IU four times a day.

**Figure 3:**
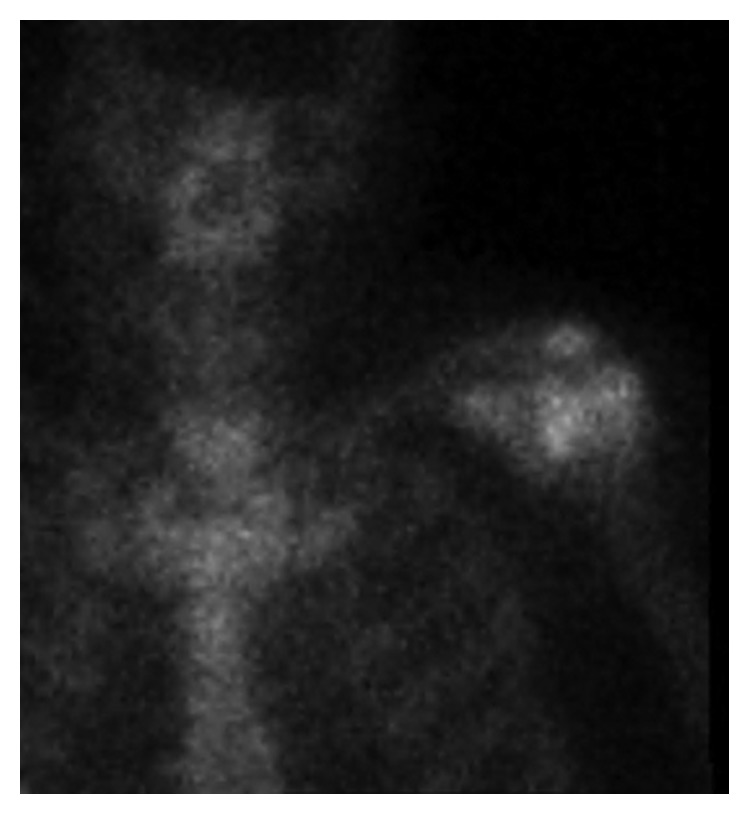
Illustration of the patient's left shoulder by Technetium-99m scintigraphy.

On day three, CRP levels were increasing as well as the pain and oedema in his left arm. On day five, arthroscopic revision of the left shoulder was performed and three biopsies were taken, but none of these cultures showed further growth of the pathogen. The rotator cuff was almost completely degenerated, and the humeral head was devoid of its hyaline cartilage. Ten days later technetium-99m bone scintigraphy showed intense activity in the left shoulder, suspicious of osteitis (Figure 3). The bone scintigraphy revealed no other focus of infection. On day 17, control transesophageal echocardiography was performed. At this time, the examination showed no suspicious signs of endocarditis, and the vegetation that was previously seen on the aortic valve could not be visualised.

The patient was sent to a dentist, which did not reveal any oral focus. The patient's clinical condition improved gradually, and he was discharged from our hospital after five weeks (36 days) with oral antibiotics (oral amoxicillin 1 g × 3 pr. day and oral rifampicin 600 mg × 2 pr. day) for further two months. In total, he received antibiotics for almost three months (87 days). During the admission, the patient lost approximately 17 kilograms in weight, primarily fluid associated with his septic condition as well as fluid from some of degree cardiac decompensation and lung stasis.

The patient was followed up in the Out-Patient Clinics for Infectious Diseases and Cardiology, which included blood samples and control transthoracic echocardiography. None of the subsequent examinations indicated signs of recurrence. The patient regained normal function in his left shoulder.

The patient reported that he had daily contact with animals including horses, though he did not recall any illness in the four horses he had taken care of the past year. He denied consuming unpasteurized milk products.

## 3. Discussion

*Streptococcus equi* subsp. *zooepidemicus* is a zoonotic pathogen, and infections of *S. equi* subsp. *zooepidemicus* in individual patients are associated with those having daily contact with animals, especially horses [1, 4, 15]. Human cases have been described with cutaneous and pulmonary entries as possible ways of transmission [13, 17], although the primary source of infection is mostly unknown, except for well-described outbreaks [3, 4, 15, 18, 19].

That the patient in the present report had infections and inflamed sores in his mouth months before admission, and concurrently had daily contact with horses, suggests the mouth as a possible entry for the zoonotic transmission. This assumption is given that the horses were infected with the bacteria, which is unknown. Therefore, it could have been interesting to obtain nasopharyngeal swabs form the horses in contact with the patient to explore the origin of infection. Even though the patient and his family denied any sign of illness from the horses, it is still possible they were infected or colonised carrier [20]. Pelkonen et al. describe three human cases of bacteraemia with *S. equi* subsp. *zooepidemicus* and identification of same bacteria in the horses, who were without any serious signs of illness [1].

The literature reveals 17 cases of endocarditis due to infection with *S. zooepidemicus* (Table 1). The Modified Duke Criteria are widely accepted for diagnostic classification of infective endocarditis (IE) [21, 22]. The diagnosis can be difficult to establish and is primarily based on blood cultures, echocardiographic findings, and clinical manifestations. In the present case, despite a strong clinical likelihood of aortic valve endocarditis based on a large mass that resolved with appropriate therapy, the patient did not meet the formal requirements for definite IE, which would be one major and three minor criteria. Given that the blood cultures were not repeated in the acute phase, bacteremia could not count as a major criterion. In addition, the rarity of *S. equi* subsp. *zooepidemicus* human infections assures that it is not considered “a typical microorganism consistent with endocarditis” which is required to constitute a major criterion. Our patient therefore fulfilled one major and two minor criteria, classifying this as a case of possible endocarditis according to the Modified Duke Criteria [21, 22]. Despite this, the patient was considered to have aortic valve endocarditis by the clinical team and was treated consistent with the presence of endocarditis, and the large mass consistent with valvular infection resolved on repeat high-resolution imaging.

An analysis from 1990 assessed the mortality rate to 40% based on six fatalities out of 15 patients [23]. Several studies have investigated a possible explanation causing a high mortality rate in affected humans with *S. equi* subsp. *zooepidemicus* [5, 9]. Salata et al. described in 1989 an increased susceptibility to disseminated infection among elderly with comorbidities, especially illnesses as chronic cardiopulmonary disease, arterial hypertension, and diabetes mellitus type II [24]. This assumption is well underpinned in the latest published data on outbreaks due to *S. zooepidemicus* [3, 18]. Additionally, published data indicate a better outcome if infected sporadically, rather than in clusters due to outbreaks [15].

Another important factor influencing the outcome is the choice of antibiotic treatment. Benzylpenicillin is preferred with bacteraemia caused by group C streptococci and can be supplemented with aminoglycoside leading to a successful result. Combining penicillins with aminoglycosides may have a positive effect of lowering the risk of needing valve replacement in the case of endocarditis [23]. In case of allergy to penicillin, previous studies have used ceftriaxone or levofloxacin combined with rifampin [4, 17, 18]. Previous studies have not found development of resistance to penicillin [3, 23].

Purulent arthritis has been described in 16 cases (Table 2). The arthroscopy was postponed to day 5 due to the patient's clinical and cardiovascular status. An arthroscopy must be conducted in a view of protecting the cartilage and is the favoured approach in treatment of purulent arthritis [25]. The results from Technetium-99m bone scintigraphy may be influenced by the surgery. A magnetic resonance imaging of the affected shoulder was not performed, although this investigation probably could give even more accurate results and affirm or debilitate the suspicious of osteitis.

## 4. Conclusion

This report highlights how virulent *S. zooepidemicus* can be when affecting humans. An 82-year-old man, who had daily contact with horses, developed a disseminated infection and was treated for sepsis, endocarditis, purulent arthritis, and osteitis. The patient responded well to the treatment, which primarily consisted of intravenous high dosage benzylpenicillin combined with gentamicin followed by oral amoxicillin and oral rifampicin. The patient recovered without permanent injuries.

It is recommended that identification of group C streptococci in humans is followed by determination of their species with the purpose of correct intervention, or in the event of an outbreak, limiting the spread of a pathogen associated with a high mortality rate. It is also of medical and epidemiological interest to identify group C streptococci at species level regarding the incidence of this virulent organism [1, 17]. In relation to invasive infection, patients in close contact with domestic animals or with a recent intake of unpasteurised dairy products, the pathogen *S. zooepidemicus* should be taken in consideration as a possible causation.

## Figures and Tables

**Table 1:** Published cases with endocarditis due to *Streptococcus equi* subsp. *zooepidemicus*.

Number	Age/sex	Year/nationality	Diagnosis	Comorbidity	Contamination	Antibiotic therapy	Pattern of resistance	Duration of treatment	Outcome	Reference
1	83/M	2016, Faroe Islands	Endocarditis, purulent arthritis, suspected osteomyelitis, sepsis	DMII, HD, IHD, low malignant prostate cancer	Horses	IV cefuroxime, benzylpenicillin, gentamicin (in total 5 days), oral amoxicillin, and rifampin	Sensitive to penicillin, ampicillin, cefotaxime, erythromycin, and clindamycin	12 weeks and 4 days	Recovered	Present case
2	59/F	2009, Canada	Meningitis, mitral endocarditis, endophthalmitis	AH, DMII, dyslipidemia, IHD, chronic renal failure, obesity, left ophthalmic vein thrombosis, ostium primum atrial septal defect, hypothyroidism, primary hyperparathyroidism and anemia	No relation to unpasteurised milk products. Her husband had daily contact with healthy horses	IV ceftriaxone and rifampin	Susceptible to all tested antibiotics according to the Clinical and Laboratory Standard Institute criteria	6 weeks	Blindness	[4]
3	70/F	2003, Spain	Pneumonia, endocarditis, bacteraemia	None	Milk-borne outbreak	Beta-lactam agent	Sensitive to penicillin, erythromycin, vancomycin, rifampin, and levofloxacin, but resistant to clindamycin and tetracycline	Unknown	Disorientation to time and place	[3]
4	73/M	Spain	Bacteraemia, endocarditis	AH, dyslipidemia, mechanical aortic valve replacement	Contact to stillborn foal two weeks before admission	Penicillin (6 weeks) and gentamicin (first 2 weeks)	Unknown	6 weeks	Recovered	[6]
5		2012, France	Bacteraemia, endocarditis	Pituitary adenoma, mechanical aortic valve	Unknown	Amoxicillin and gentamicin	Sensitive to beta-lactamins, macrolides, chloramphenicol, co-trimoxazole, glycopeptides, and rifampin, but resistant to tetracycline	6 weeks	Recovered	[7]
6	79/M	2010, England	Bacteraemia, endocarditis	None	Horse manure	Benzylpenicillin and vancomycin	Unknown	6 weeks	Recovered	[8]
7	57/M	2011, Finland	Sepsis, meningitis, endocarditis	Aortic valve insufficiency	Horses	Penicillin (5 weeks) and gentamicin (10 first days)	Sensitive to erythromycin, clindamycin, penicillin, vancomycin, and cephalexin	5 weeks	Recovered	[1]
8	58/F	1980's, China	Bacteraemia, endocarditis	None	No relation to horses or unpasteurised milk	Benzylpenicillin	Unknown	Unknown	Recovered	[9]
9	51/M	1982, unknown	Bacteraemia, endocarditis	Rheumatic HD	Unknown, but was a farmer	IV penicillin (4 weeks) and IM streptomycin (2 weeks)	Unknown	4 weeks	Recovered	[10]
10	52/F	1984, England	Sepsis, bacteraemia, endocarditis	Unknown	A milk-borne outbreak	Penicillin and cephalosporin	Unknown	4 weeks	Impairment of peripheral flow in one arm	[11]
11	73/M	1984, England	Sepsis, bacteraemia, endocarditis, meningitis	IHD	A milk-borne outbreak	Unknown	Unknown	Unknown	Died	[11]
12	79/M	1984, England	Sepsis, bacteraemia, endocarditis		A milk-borne outbreak	Cephalosporin, ampicillin, and metronidazole	Unknown	2 weeks	Died	[11]
13	81/M	1979, England	Bacteraemia, endocarditis, cholecystitis	Chronic rheumatic HD	Unknown	Oral ampicillin, IV benzyl penicillin (3 weeks and 3 days) and gentamicin (first 10 days), changed to oral amoxicillin (2 weeks)	Unknown	5 weeks and 3 days	Died	[12]

The table includes 12 of the 17 cases, exclusive the present cases. The last three cases are not included because the information in the articles was either impossible to get access to or not detailed enough to fulfil the table: two cases of endocarditis due to *S. zooepidemicus* from the article “*Endocarditis due to group C streptococci according to Group C streptococcal bacteremia: analysis of 88 cases*.” One case in *“Prolonged fever Streptococcus equi spp. zooepidemicus (endocarditis aortic complicated with mycotic aneurysm infrarenal)*.” The article “*Group C streptococcal infections associated with eating home cheese–New Mexico”* describes one case with endocarditis. AH: arterial hypertension; IHD: ischemic heart disease; HD: heart disease; DMII: diabetes mellitus type II; IV: intravenous; IM: intramuscular injection.

**Table 2:** Published cases with septic arthritis due to *Streptococcus equi* subsp. *zooepidemicus*.

Number	Age/sex	Year/nationality	Diagnosis	Comorbidity	Contamination	Antibiotic therapy	Pattern of resistance	Duration of treatment	Outcome	Reference
1	60/M	2008, Germany	Purulent arthritis	Chronic back pain	Horses (cutaneous wound)	IV amoxicillin with clavulanic acid, gentamicin	Tetracycline	5 weeks and 4 days	None	[13]
2	72/F	2003, Spain	Septic arthritis	None	Milk-borne outbreak	Beta-lactam agent	Sensitive to penicillin, erythromycin, vancomycin, rifampin, and levofloxacin, but resistant to clindamycin and tetracycline	Unknown	Limited mobility	[3]
3	79/M	2003, Spain	Bacteraemia and septic arthritis	DMII, AH, HD	Milk-borne outbreak	Beta-lactam agent	Same as case number 3	Unknown	Recovered	[3]
4	62/M	2011, Finland	Bacteraemia septic arthritis (in two joints)	DMII,	Horses	Cefuroxime (first three days), changed to IV vancomycin (unknown duration), changed to penicillin G with clindamycin (2 weeks), changed to oral cephalexin and clindamycin for 1 week	Sensitive to erythromycin, clindamycin, penicillin, vancomycin, and cephalexin	Unknown, but >3 weeks	Recovered	[1]
5	70/M	1985, England	Septic arthritis, sepsis, acute renal failure	AH, HD, osteo- and rheumatoid type arthropathy	No daily animal contact, possible ingested unpasteurised milk	IV penicillin G (3 week), oral penicillin (3 months)	Unknown	3 months and 3 weeks	Recovered	[14]
6	78/M	2003–2015, Sweden	Sepsis, and septic arthritis	AH, HD, CLL	Unknown			12 weeks and 8 days	Unknown	[15]
7	71/M	2003–2015, Sweden	Septic arthritis	WM	Unknown	Penicillin G followed by amoxicillin	Unknown	17 weeks and 1 day	Unknown	[15]
8	79/M	2003–2015, Sweden	Septic arthritis	AH	Horses	Penicillin G followed by amoxicillin	Unknown	17 weeks and 1 day	Unknown	[15]
9	42/M	Unknown	Septic arthritis	None	Horses	Penicillin G	Unknown	Unknown	Recovered	[16]
10	74/F	1992, USA	Septic arthritis, bacteraemia	None	No relation to contact with animals	Penicillin changed to vancomycin due to suspicion of penicillin allergy	Unknown	5 weeks	Recovered	[16]

The table includes 10 of the 16 cases. The last six cases are not included because the information in the articles was either impossible to get access to or not detailed enough to fulfil the table: One case of purulent arthritis in the article “*An outbreak of Streptococcus equi subspecies zooepidemicus associated with consumption of fresh goat cheese.*” Three more cases are described in “*Group C streptococcal bacteremia: analysis of 88 cases*.” Finally, two cases from England presented in “*Group C streptococci in human infection—a study of 308 isolates with clinical correlations*.” AH: arterial hypertension; IHD: ischemic heart disease; HD: heart disease; DMII: diabetes mellitus type II; WM: Waldenströms macroglobulinemia; CLL: chronic lymphatic leukaemia; USA: the United States of America.
